# Unilateral biportal endoscopic decompression for thoracic intraspinal gout with ossified ligamentum flavum: a case report and literature review

**DOI:** 10.3389/fsurg.2026.1831917

**Published:** 2026-06-03

**Authors:** Fangling Zhong, Chenxing Huang, Xiaoteng Feng, Zhaojun Cheng, Wenjing Su, Weibo Yu, Hui Ren, Binwei Chen, Xiaobing Jiang

**Affiliations:** 1 Department of Spine Surgery, The Second Affiliated Hospital of Guangzhou Medical University, Guangzhou, Guangdong, China; 2 The Second Clinical College of Guangzhou Medical University, Guangzhou, Guangdong, China

**Keywords:** gouty tophus, minimally invasive surgery, ossification of the ligamentum flavum, spinal stenosis, unilateral biportal endoscopy

## Abstract

**Objective:**

Intraspinal tophaceous gout is rare but may cause severe neurological deficits when it compresses the spinal cord. Thoracic spinal stenosis due to ossification of the ligamentum flavum (OLF) is an additional, common dorsal compressive pathology. We report a patient with thoracic spinal canal stenosis due to OLF with concomitant epidural tophaceous gout, successfully treated using unilateral biportal endoscopy (UBE) combined with percutaneous pedicle screw fixation.

**Clinical presentation and intervention:**

A 50-year-old man with a 4-year history of gout and multiple peripheral tophi presented with a 2-year history of back pain that worsened over 2 weeks, accompanied by bilateral lower-limb pain, numbness, and marked weakness (lower-limb strength grade 2). Preoperative imaging demonstrated severe thoracic canal stenosis at T10-T11 due to OLF and new compression fractures at T8 and T10. Laboratory tests revealed leukocytosis, elevated ESR and CRP, and hyperuricemia. The patient underwent UBE-assisted decompression with resection of the OLF at T10-T11, followed by percutaneous pedicle screw fixation at T7-T9 and T11-T12. Intraoperatively, abundant chalky-white tophaceous material was identified in the epidural space and confirmed histologically as monosodium urate deposits with granulomatous inflammation. His symptoms gradually resolved, muscle strength improved, and he achieved good functional recovery one year post-operatively.

**Conclusion:**

This case suggests that UBE may be a precise minimally invasive option for selected patients with thoracic intraspinal gout and underscores the necessity of multidisciplinary care and long-term urate-lowering therapy.

## Introduction

Gout is a metabolic disorder caused by abnormalities in uric acid metabolism, leading to hyperuricemia and the deposition of monosodium urate crystals. Pathological changes primarily involve bone destruction in affected joints and fibrosis of surrounding soft tissues (1). The global incidence of gout has risen in recent years, with a prevalence of approximately 3.9% among U.S. adults, affecting about 8.3 million individuals (2). Clinically, gouty tophi typically manifest as subcutaneous nodules, most commonly located at the first metatarsophalangeal joint, dorsum of the foot, or heel, though other peripheral joints may also be involved (1). However, tophaceous deposition within the spinal canal is relatively rare. Since Kersley et al. first described spinal gout in 1950, only a limited number of cases have been reported in the literature (3). The onset is usually insidious, and most patients remain asymptomatic until tophi compress the spinal cord or nerve roots, resulting in sensory or motor deficits, or in severe cases, sphincter dysfunction. The appearance of such neurological symptoms indicates advanced disease progression. These manifestations are typically refractory to conservative management and often necessitate surgical intervention to relieve neural compression and preserve function.

For patients with neurological deficits caused by intraspinal tophaceous compression, open laminectomy is currently the standard treatment (4, 5). However, this procedure involves extensive dissection and exposure, leading to significant surgical trauma and increased perioperative risks. With advancements in minimally invasive spine surgery, percutaneous endoscopic techniques have been increasingly applied for tophus removal in the cervical and lumbar regions, demonstrating favorable outcomes (6, 7). Unilateral biportal endoscopy (UBE), an emerging minimally invasive technique, has recently gained attention for its distinct advantages. UBE employs two independent portals for visualization and instrumentation, preserving the benefits of minimally invasive surgery while providing an expanded and clearer surgical field. The enhanced magnification and superior visibility make the UBE system particularly valuable for managing anatomically complex or rare lesions such as intraspinal tophi. To the best of our knowledge, reports of thoracic OLF coexisting with intraspinal gouty tophus treated using UBE are scarce. We present a case managed with UBE decompression combined with percutaneous pedicle screw fixation, which resulted in neurological improvement at 1-year follow-up.

## Case presentation

A 50-year-old man (height 167 cm, weight 90 kg; BMI 32.3 kg/m^2^) was admitted with a 2-year history of back pain that worsened over the preceding 2 weeks, accompanied by bilateral lower-limb pain, numbness, and weakness refractory to conservative treatment (physical therapy and NSAIDs). Past history included gout for 4 years with multiple peripheral tophi, hypertension, and type 2 diabetes mellitus. Two months prior to admission, he underwent excision of tophi from the left middle finger, dorsum of the hand, and elbow. He denied trauma, fever, chills, or night sweats. On admission he was transported on a gurney. Physical examination revealed restricted thoracic motion and marked tenderness over the thoracic spinous processes and paraspinal muscles. Neurological assessment demonstrated grade 5 muscle strength in the upper limbs but grade 2 in both lower limbs. The straight-leg raising test was negative. The Babinski sign was positive bilaterally. Neurological status was evaluated using the modified Japanese Orthopaedic Association (mJOA) score for thoracic myelopathy (maximum 11 points). The preoperative mJOA score was 5, and the back visual analog scale (VAS) score was 8, leg VAS was 7.

**Figure 1:**
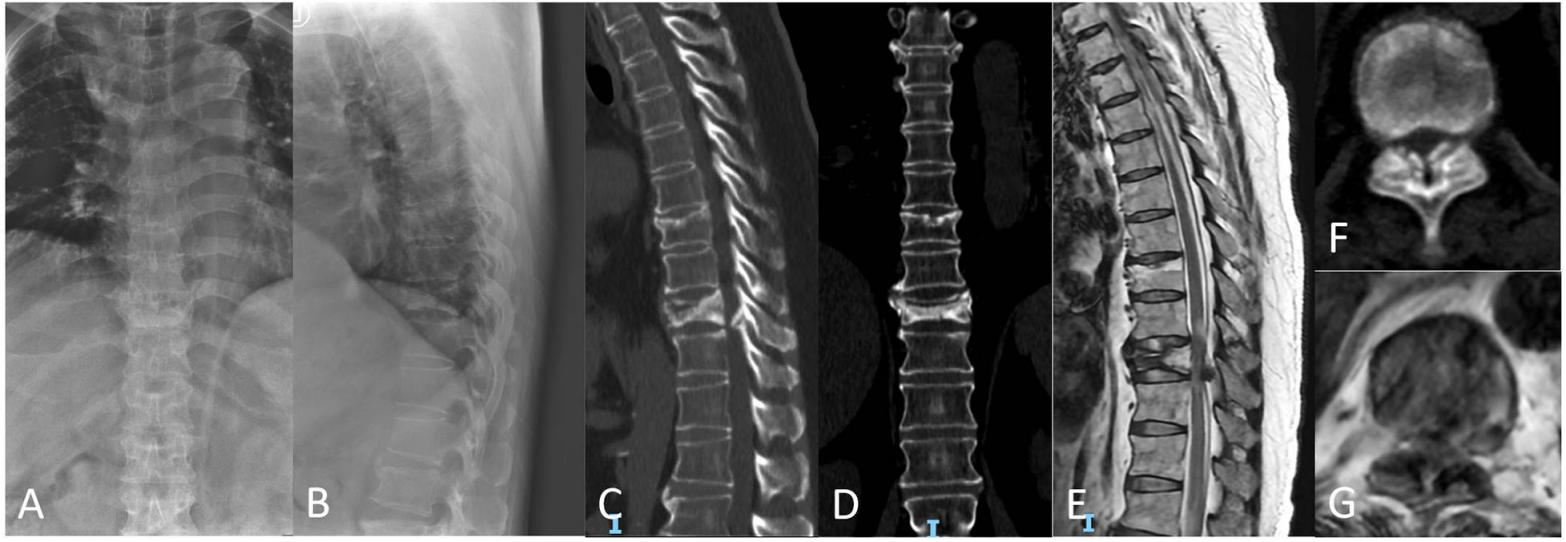
Anteroposterior **(A)** and lateral **(B)** radiographs demonstrating thoracic compression fracture and degenerative changes. Sagittal **(C)** and coronal **(D)** views of CT and MRI **(E)** scans revealing ossification of the ligamentum flavum at the T10-T11 level with associated thoracic spinal canal stenosis, as well as new compression fractures of the T8 and T10 vertebral bodies. Axial CT **(F)** and MRI **(G)** images at the T10-T11 level further illustrating the extent of ossification and resultant spinal canal narrowing.

Plain radiographs demonstrated thoracic compression fracture and degenerative changes (Figures 1A,B). Computed tomography (CT) and magnetic resonance imaging (MRI) revealed new compression fractures of T8 and T10 and a large ossified lesion consistent with OLF at T10-T11, occupying most of the spinal canal with severe thoracic stenosis (Figures 1C–G). Due to the atypical radiologic appearance resembling OLF, an intraspinal tophus was not initially suspected. Laboratory investigations showed leukocytosis (12.8 × 10^9^/L; reference 3.5–9.5 × 10^9^/L), elevated serum uric acid (533 µmol/L; reference 208–428 µmol/L), elevated C-reactive protein (CRP) at 29.17 mg/L (reference 0–6 mg/L), elevated erythrocyte sedimentation rate (ESR) at 120 mm/h (reference 0–20 mm/h), slightly elevated procalcitonin (PCT) at 0.1 ng/mL (reference 0–0.046 ng/mL). Bone mineral density testing indicated osteoporosis (T-score −2.6). No significant abnormalities were identified in the cervical or lumbar spine. The preoperative diagnoses included thoracic spinal canal stenosis (T10-T11), OLF (T10-T11), compression fractures (T8, T10), bilateral lower limb paraparesis, history of chronic gout, osteoporosis, hypertension, and diabetes mellitus.

## Surgical technique

**Figure 2:**
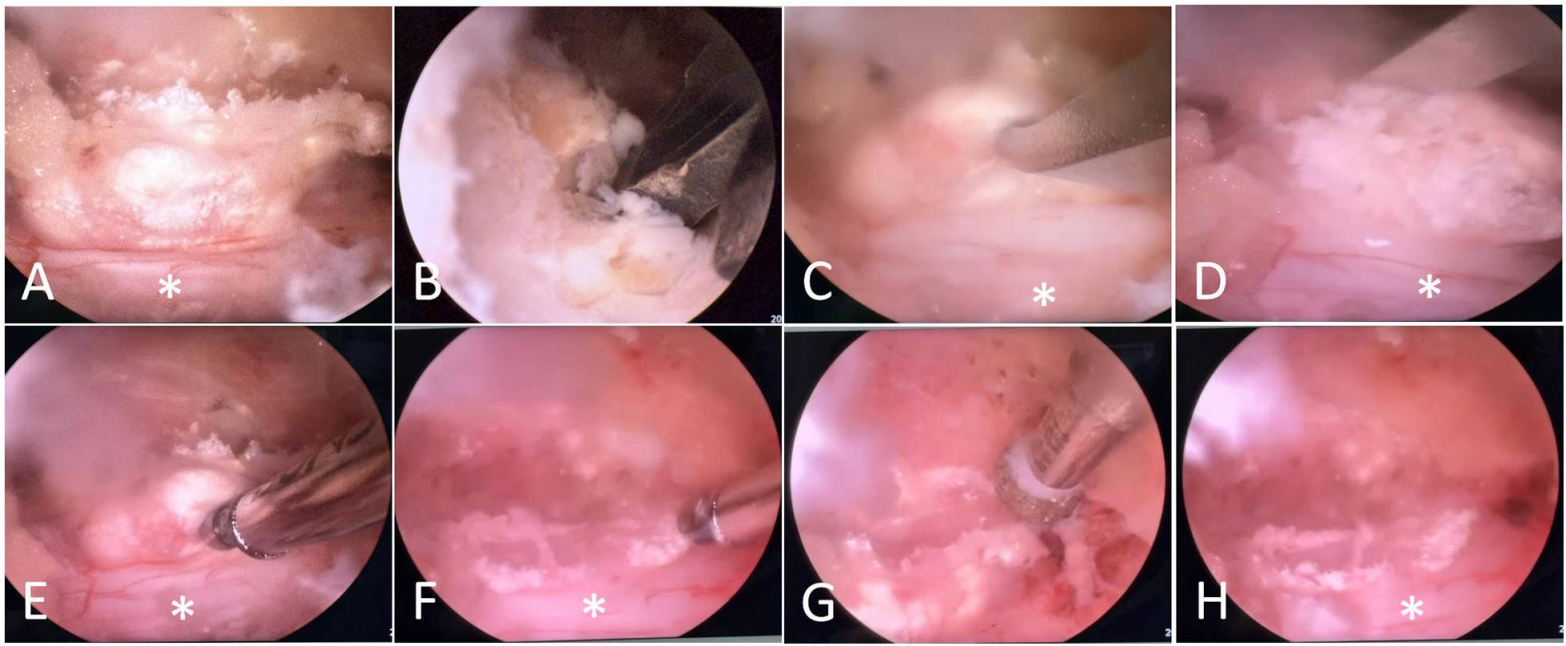
Intraoperative endoscopic views of OLF during UBE decompression. **(A)** Chalky-white tophaceous material was identified in the epidural space, together with hypertrophied/ossified ligamentum flavum tissue contributing to dorsal compression. **(B–D)** Piecemeal removal of epidural tophaceous tissue and ligamentum flavum using forceps and dissector. **(E,F)** Bipolar radiofrequency coagulation for hemostasis. **(G)** High-speed diamond burr used for controlled thinning/removal of ossified ligamentum flavum. **(H)** Adequate decompression with restoration of dural pulsation. * Indicates the nerve.

The procedure was performed under general anesthesia with the patient in the prone position. After standard sterilization and draping, the target spinal levels were confirmed, and skin incision sites were marked under C-arm fluoroscopic guidance. The surgeon, positioned on the patient's right side, made two approximately 1-cm longitudinal incisions—one at the lower margin of the cephalad lamina and the other at the upper margin of the caudal lamina—approximately 0.5 cm from the midline to facilitate contralateral visualization and instrument manipulation. Sequential dilation was performed to expose the lamina, and separate endoscopic viewing and working portals were established. Continuous saline irrigation was used to maintain a clear operative field. Under endoscopic visualization and guided by preoperative imaging, the ossified margins of the ligamentum flavum were delineated. Stepwise laminotomy was then carried out using a high-speed drill to remove the ossified segments. We did not perform conventional open laminectomy or facetectomy. Instead, UBE-assisted unilateral laminotomy with bilateral decompression was performed. The ossified ligamentum flavum was thinned and removed stepwise using a high-speed drill and pituitary rongeurs under continuous endoscopic visualization. The facet joints and posterior ligamentous complex were preserved as much as possible to maintain thoracic stability. Intraoperatively, abundant chalky-white material was identified in the epidural space (Figure 2A). Using grasping forceps, a nerve dissector, and bipolar radiofrequency, the abnormal tissue and residual ligamentum flavum were excised in fragments. The adherent tophaceous material was removed piecemeal with minimal traction on the dura and spinal cord, and the ossified region was meticulously debrided with pituitary rongeurs. Complete hemostasis was achieved with radiofrequency coagulation. After bilateral decompression, endoscopic inspection confirmed satisfactory dural pulsation and sufficient space around the nerve roots (Figures 2B–H). During the surgery, no purulent material was observed. A drainage tube was placed. The UBE decompression phase lasted approximately one hour, with an estimated blood loss of less than 50 mL.

Subsequently, while the patient remained in the prone position, percutaneous pedicle screw fixation was performed at the T7, T8, T9, T11, and T12 levels, skipping the fractured T10 vertebra to ensure optimal screw purchase strength. Under C-arm fluoroscopic guidance in anteroposterior and lateral views, the pedicle projections from T7 to T12 were identified and marked. Small longitudinal incisions (approximately 1.2 cm) were made, and the paraspinal muscles were bluntly dissected to expose the lateral facets. Guidewires were inserted sequentially, and after confirming appropriate trajectory and depth, pedicle screws were placed. Given the patient's osteoporosis, cement-augmented fenestrated pedicle screws were used at the terminal levels (T7 and T12) to improve screw purchase and construct stability in this short-segment fixation. Bone cement was injected through the pedicle screws under continuous fluoroscopic monitoring. No cement leakage or venous extravasation was observed. Final fluoroscopy confirmed proper screw positioning and uniform cement distribution. Pre-contoured connecting rods were inserted subcutaneously and secured with locking nuts. The surgical field was thoroughly irrigated, and all incisions were closed in layers. Intraoperative neurophysiological monitoring, including motor-evoked potentials and somatosensory-evoked potentials, was used throughout the decompression and fixation procedures to reduce the risk of iatrogenic neurological injury. The total operative time was approximately three hours, with an estimated blood loss of 200 mL, and no transfusion was required.

## Outcome

**Figure 3:**
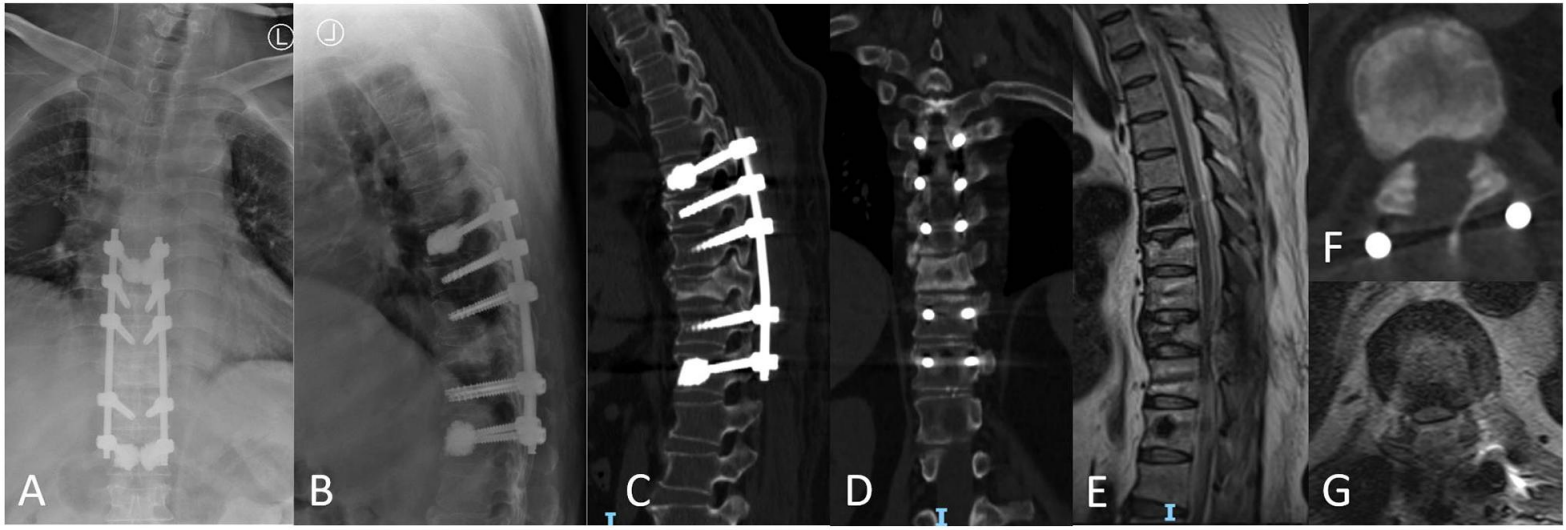
Postoperative imaging. Anteroposterior **(A)** and lateral **(B)** radiographs demonstrating stable pedicle screw fixation with bone cement augmentation at T7 and T12, maintaining proper thoracic alignment. Sagittal **(C)** and coronal **(D)** views of CT and MRI **(E)** scans demonstrating complete removal of the ossified ligamentum flavum at the T10-T11 level and adequate decompression of the spinal canal. Axial CT **(F)** and MRI **(G)** images confirming total excision of the ossified lesion and satisfactory decompression of the spinal cord.

**Figure 4:**
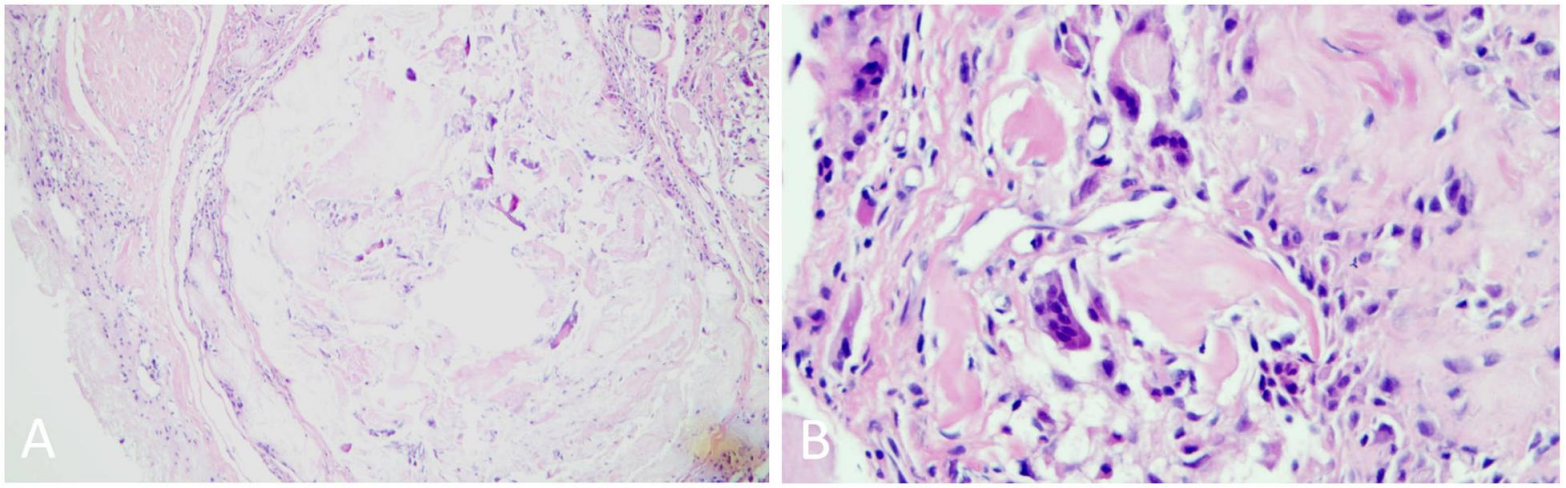
Histopathological findings of the excised lesion. Hematoxylin and eosin (H&E) staining reveals deposits of monosodium urate crystals surrounded by fibrous tissue, fibroblasts, lymphocytes, and multinucleated giant cells, consistent with tophaceous gout. **(A)** Overall view. **(B)** Enlarged view.

Postoperatively, the patient received neurotrophic therapy and initiated early bilateral lower-limb rehabilitation exercises. Postoperative radiographs demonstrated stable instrumentation and preserved thoracic alignment (Figures 3A,B). CT and MRI confirmed complete removal of the ossified ligamentum flavum at T10-T11 with effective decompression of the spinal canal (Figures 3C–G). His back pain, numbness, and weakness gradually improved, and no perioperative complications, such as cerebrospinal fluid leakage, infection, or delirium, were observed. Intraoperative bacterial and fungal cultures were performed, and the results were negative for both pathogens, confirming the absence of infection. Histopathological examination of the resected tissue revealed monosodium urate crystal deposits surrounded by fibroblasts, lymphocytes, and multinucleated giant cells with granulomatous inflammation, confirming the diagnosis of gouty tophus (Figure 4). The patient was referred to Rheumatology for urate-lowering therapy (febuxostat) and flare prophylaxis (colchicine). An anti-osteoporotic regimen (calcium, vitamin D, denosumab) was also initiated. Clinical outcomes were assessed using the thoracic mJOA score and VAS at discharge, 6 months, and 1 year postoperatively. At discharge, the mJOA score improved to 7, back VAS decreased to 4, and leg VAS decreased to 4. At the six-month follow-up, the mJOA score reached 8, back VAS decreased to 2, and leg VAS decreased to 3, and lower-limb muscle strength improved to grade 4. By the one-year follow-up, the mJOA score improved to 9. Back and leg VAS scores dropped to 0 and 1, respectively, while lower-limb muscle strength stabilized at grade 4. Residual mild lower-limb weakness persisted at 1 year, with no further improvement in muscle strength after the 6-month follow-up. No recurrence or symptom exacerbation was detected. Long-term follow-up imaging was not repeated in view of clinical stability and satisfactory neurological recovery. This study was approved by the Medical Ethics Committee of the Second Affiliated Hospital of Guangzhou Medical University, and written informed consent for publication of the case details and associated images was obtained from the patient.

## Discussion

Gout is a metabolic disorder with a relatively low incidence of spinal involvement (8). However, spinal gouty tophi are frequently misdiagnosed as disc herniation, tumors, or infections, potentially leading to delayed treatment and irreversible deficits. Thoracic intraspinal gout is exceedingly rare compared to lumbar involvement. Given the narrow thoracic canal, even small deposits can cause significant cord compression. Currently, there is no standardized treatment protocol for this condition. According to previous studies, patients presenting with spinal cord or nerve root compression are typically managed with open decompression, accompanied by fusion and fixation when necessary. Although back pain represents the most frequent clinical manifestation of spinal gout, the occurrence of paraplegia or other neurological deficits indicates spinal cord or nerve root compression and necessitates urgent surgical decompression (8). Nevertheless, open surgery often requires extensive soft-tissue dissection and may be associated with substantial intraoperative blood loss and a longer postoperative recovery, which can be particularly challenging for patients with gout who frequently have multiple systemic comorbidities (9).

Beyond surgical strategy, this case also emphasizes the importance of differential diagnosis in patients presenting with rapid neurological deterioration. Acute or subacute paraparesis or paraplegia with back pain may result from diverse compressive pathologies, including epidural abscess, spinal tumors, metastatic disease with pathological fracture, hematoma, disc herniation, ossification of spinal ligaments, and metabolic crystal deposition diseases. Recent literature on gout-related bilateral carpal tunnel syndrome has shown that tophaceous deposition may cause clinically significant peripheral nerve compression, supporting the concept that advanced gout can present as a systemic compressive neuropathy rather than merely a peripheral joint disorder (10). In addition, although etiologically distinct from the present case, acute paraplegia caused by melanoma-related spinal metastasis and pathological vertebral fracture provides an important comparator for rapidly progressive spinal cord compression and highlights the need to distinguish metabolic, infectious, and neoplastic causes in urgent clinical settings (11). In the present patient, the history of chronic gout with multiple peripheral tophi, hyperuricemia, intraoperative chalky-white deposits, negative cultures, and histopathological confirmation of monosodium urate deposition collectively supported the final diagnosis of thoracic intraspinal tophaceous gout coexisting with OLF.

In this case, gouty tophi were successfully excised using a UBE approach through two approximately 1-cm incisions, and the patient experienced an uneventful recovery. This case suggests that UBE may be a feasible minimally invasive option for thoracic intraspinal gout, with potential advantages in visualization, targeted decompression, and postoperative recovery. The complexity of the present case was heightened by the coexistence of intraspinal tophi with T8 and T10 vertebral compression fractures. The T8 and T10 compression fractures were interpreted as recent osteoporotic insufficiency fractures rather than traumatic fractures, because the patient denied any history of trauma and bone mineral density testing demonstrated osteoporosis with a T-score of −2.6. Chronic gout, systemic inflammation, diabetes, obesity, and reduced mobility may have further contributed to impaired bone quality. No clinical, microbiological, or postoperative evidence suggested infectious spondylitis or malignant pathological fracture in this patient. A single-stage procedure combining UBE-assisted decompression with percutaneous pedicle screw fixation achieved both adequate neural decompression and stable spinal reconstruction. Postoperative imaging showed satisfactory fracture reduction and spinal alignment, further suggesting the feasibility of this combined minimally invasive strategy in selected patients.

The principal innovation of this study lies in extending the application of the established UBE technique to the challenging management of thoracic intraspinal gouty tophus (12). Building upon prior experience in treating thoracic ossification of the ligamentum flavum, the dual-portal configuration provides a magnified surgical field that allows for fine visualization and meticulous dissection of irregular, whitish tophaceous deposits adherent to neural structures. Furthermore, the UBE approach enables bilateral decompression via a unilateral route, thereby achieving complete spinal cord decompression while preserving key posterior stabilizing structures, including the supraspinous and interspinous ligaments and facet joints (13). This principle of maximal decompression with minimal structural disruption is particularly critical in the thoracic spine, where maintaining physiological stability is essential.

Xie et al. reported a rare case of cervical ligamentum flavum gouty tophus treated with posterior percutaneous endoscopic decompression, achieving good neurological recovery (6). Similarly, Chen et al. described lumbar intraspinal tophaceous gout managed with percutaneous transforaminal endoscopic decompression (PTED), with excellent postoperative outcomes (7). These reports suggest that percutaneous endoscopic surgery may be a safe and minimally invasive option for selected cases of spinal gout. Compared with open surgery, UBE offers distinct advantages, including reduced intraoperative blood loss, less postoperative pain, shorter hospitalization, and faster functional recovery—features particularly beneficial for elderly patients with multiple comorbidities (14). This case extends the application of UBE to thoracic intraspinal gout coexisting with OLF. The dual-portal configuration offers a magnified field and improved instrument triangulation, enabling controlled bone windowing and safe removal of adherent tophi while preserving posterior stabilizers (15). Compared with single-portal techniques such as PTED—which are better suited for ventral lesions—UBE provides wider exposure for dorsal pathologies typical of OLF or dorsal tophi (16). In our patient, combining UBE decompression with short-segment fixation achieved neural decompression and mechanical stability in a single stage. Because the patient had osteoporosis, cement augmentation was applied at the terminal vertebrae to mitigate the risk of screw loosening and to enhance fixation strength.

From an imaging perspective, the present case further illustrates the diagnostic difficulty of spinal gout. In this patient, the epidural tophaceous component radiologically mimicked ossification of the ligamentum flavum and was not definitively diagnosed preoperatively. Therefore, the diagnosis of epidural gouty tophus was established mainly on the basis of intraoperative visualization of chalky-white deposits and histopathological examination demonstrating monosodium urate crystal deposition with granulomatous inflammation. Although preoperative inflammatory markers (including PCT and CRP) were elevated, the intraoperative absence of purulence and negative culture results effectively excluded an infectious etiology, indicating that the systemic inflammatory response was likely secondary to the acute gouty flare. Dual-energy computed tomography (DECT), which differentiates tissues based on chemical composition, has demonstrated high sensitivity and specificity for identifying urate crystal deposits. However, its diagnostic accuracy can be affected by vascular calcifications and motion artifacts. Furthermore, DECT is most commonly utilized in patients with a known history of gout and offers limited differential diagnostic value for paraspinal lesions lacking characteristic clinical manifestations (17). Despite its potential advantages, the widespread clinical application of DECT remains restricted by equipment availability and cost considerations. Therefore, in patients presenting with the triad of unexplained back pain, neurological dysfunction, and a paraspinal mass, spinal gouty tophus should be included in the differential diagnosis. A multidisciplinary assessment integrating MRI features with serum uric acid evaluation is essential to improve diagnostic accuracy and reduce the likelihood of misdiagnosis (18).

The UBE technique offers both diagnostic and therapeutic advantages. It enables real-time visualization and targeted biopsy for histopathological confirmation while simultaneously providing prompt and adequate neural decompression. Intraoperatively, particular caution should be taken to avoid excessive traction or sharp dissection of tophaceous tissue adherent to the dura. The primary surgical objective should be adequate decompression rather than complete excision, as aggressive removal may increase the risk of dural tears and cerebrospinal fluid leakage. Endoscopic magnification facilitates precise bone window enlargement and controlled lesion removal using pituitary rongeurs, nerve dissectors, and bipolar radiofrequency instruments, followed by meticulous hemostasis. This approach may facilitate controlled lesion removal under direct visualization while minimizing the risk of dural injury when performed by experienced surgeons.

Given the high risk of postoperative acute gout flares—with reported incidence rates of up to 44% in one cohort—systematic perioperative management is essential (19). Close collaboration with rheumatologists is crucial to establish individualized pharmacologic prophylaxis, typically involving colchicine or NSAIDs/cyclooxygenase-2 (COX-2) inhibitors to prevent acute attacks. Long-term urate-lowering therapy, such as allopurinol or febuxostat, should be initiated to maintain serum uric acid levels below 300–360 μmol/L (20). Multidisciplinary coordination is therefore indispensable to prevent recurrence, minimize complications, and achieve sustained clinical improvement (21, 22).

This single-case, retrospective report with short follow-up limits generalizability and assessment of recurrence risk. Selection bias is inherent, and the findings may not be generalizable to other patient populations or lesion patterns. In addition, preoperative DECT was not performed, and the follow-up duration (1 year) may be insufficient to fully assess recurrence, delayed instability, or late complications. Prospective multicentre studies are needed to validate these findings. Further work should refine non-invasive diagnostics (for example, broader clinical use of DECT or PET-CT) and investigate mechanisms of spinal tophus formation and progression.

## Conclusion

The UBE technique may represent a feasible minimally invasive approach for managing thoracic intraspinal gouty tophi. Heightened clinical awareness is essential for accurate diagnosis. Optimal outcomes depend on precise surgical intervention integrated with comprehensive long-term medical management and multidisciplinary collaboration.

## Data Availability

The original contributions presented in the study are included in the article/Supplementary Material, further inquiries can be directed to the corresponding author/s.
